# Mammalian Inner Ear-Resident Immune Cells—A Scoping Review

**DOI:** 10.3390/cells13181528

**Published:** 2024-09-12

**Authors:** Betül Karayay, Heidi Olze, Agnieszka J. Szczepek

**Affiliations:** 1 Department of Otorhinolaryngology, Head and Neck Surgery, Charité—Universitätsmedizin Berlin, Corporate Member of Freie Universität Berlin and Humboldt-Universität zu Berlin, 10117 Berlin, Germany; betuel.karayay@charite.de (B.K.); heidi.olze@charite.de (H.O.); 2 Faculty of Medicine and Health Sciences, University of Zielona Góra, 65-046 Zielona Góra, Poland

**Keywords:** inner ear, macrophages, mast cells, lymphocytes, steady-state tissues, inner ear homeostasis

## Abstract

Background: Several studies have demonstrated the presence of resident immune cells in the healthy inner ear. Aim: This scoping review aimed to systematize this knowledge by collecting the data on resident immune cells in the inner ear of different species under steady-state conditions. Methods: The databases PubMed, MEDLINE (Ovid), CINAHL (EBSCO), and LIVIVO were used to identify articles. Systematic reviews, experimental studies, and clinical data in English and German were included without time limitations. Results: The search yielded 49 eligible articles published between 1979 and 2022. Resident immune cells, including macrophages, lymphocytes, leukocytes, and mast cells, have been observed in various mammalian inner ear structures under steady-state conditions. However, the physiological function of these cells in the healthy cochlea remains unclear, providing an opportunity for basic research in inner ear biology. Conclusions: This review highlights the need for further investigation into the role of these cells, which is crucial for advancing the development of therapeutic methods for treating inner ear disorders, potentially transforming the field of otolaryngology and immunology.

## 1. Introduction

The inner ear is a multifaceted organ that provides the sense of hearing and balance necessary for the proper functioning of the central auditory and vestibular systems [1,2]. In addition to connecting the organism to the environment, it is also responsible for mediating social interactions [3]. The neuroanatomical and histological description of the inner ear usually focuses on its critical elements: the sensory epithelium and respective innervation [4,5], as well as cells that support the functional properties of the inner ear, such as pillar, Deiters’, Claudius’, or Hensen’s cells in the cochlea, the specialized cells of the stria vascularis producing endolymph for the entire inner ear, or the vestibular supporting cells producing otogelin and otoanchorin, both extracellular matrix proteins necessary for the proper function of the inner ear [5,6].

The term “immune-privileged tissue”, first coined in the forties of the last century and referring to the fact that such tissues do not reject a graft [7,8], has long been applied to the inner ear. This was done even though there was no precedent for tissue transplantation into the inner ear, but there was the blood–labyrinth barrier, similar to the blood–brain barrier [9]. The long-discussed existence of an inner ear lymphatic system was clarified by demonstrating rapid antigen drainage from the inner ear to the superficial ventral cervical lymph nodes of the guinea pig, as well as antigen uptake by cochlear resident macrophages and fibrocytes [10]. Over time, the presence of immune cells or immune mediators in the inner ear has been linked to pathological conditions, including infections caused by pathogens [11] or inflammation due to factors such aging [12] or drug exposure [13]. The initial electron microscopy studies have demonstrated the existence of macrophages within the cochlea of noise-exposed guinea pigs [14]. This discovery was corroborated by additional reports of infiltrating and resident macrophages in the cochlea and a modest resident population of CD45^+^ cells in the inferior region of the spiral ligament and spiral wall [15]. Furthermore, observations have been made regarding the increased expression of immune mediators, including cytokines, chemokines, and cell adhesion molecules, which have been shown to result in an influx of immune cells into the inner ear. Accordingly, it has been postulated that cells of hematopoietic origin and the mediators they produce may play a pivotal role in the damage to hair cells or neurons, ultimately resulting in hearing loss [15,16].

Compared to the cell biology, biophysics, and genetics of the inner ear, the immunology of the inner ear has been the subject of relatively limited investigation and warrants further study in this area. Our interest was to define the cellular component of the immune system residing in the inner ear under normal physiological conditions. Therefore, we undertook a comprehensive review of the existing evidence to advance our understanding of this area of research. We used a scoping review methodology to delineate the evidence for resident immune cells in the inner ear of different species under steady-state conditions. In addition, we aimed to summarize the cell types that have been the focus of these studies and to identify gaps in the existing literature. Therefore, the primary objectives were to identify the types of immune cells present in the mammalian inner ear under steady-state conditions and to determine the distribution of these cells across species. Consequently, the goal was to gain new knowledge that may facilitate the development of advanced therapeutics for inner ear disorders.

## 2. Materials and Methods

### 2.1. Protocol

To draft our protocol and scoping review, we followed the described methodology by Arksey and O’Malley [17] and the Preferred Reporting Items for Systematic Reviews and Meta-Analyses extension for Scoping Reviews (PRISMA-ScR) guidelines for scoping reviews [18]. The research team revised the protocol. The final version of the detailed protocol is accessible in Supplementary File S1. A detailed search strategy for all databases used is documented in Supplementary File S2. When the study was first drafted in 2019, and before the search was conducted, we attempted to register it in PROSPERO; however, due to the scoping nature of this review, such registration was not possible.

### 2.2. Information Sources and Search Strategy

A systematic search was performed in electronic databases, including PubMed, MEDLINE (Ovid), CINAHL (EBSCO), and LIVIVO. The authors designed the search strategy collaboratively, with B.K. taking the lead. The first author (B.K.) initially performed the search on 23 April 2020. The search was repeated on 22 August 2023, to avoid missing articles published after the search date. Matching search terms were used for each database, including free text words, MeSH terms, and Boolean operators. Detailed information about each database, search dates, restrictions or filters applied, number of records retrieved (for each database), and search terms for each electronic database were recorded. The search was limited by language (English and German) but not by year or study design. The reference lists of included articles were screened for missing studies that were relevant but not included in our search. A comprehensive search was conducted to minimize the possibility of missing studies that may be relevant to our research objectives. The final search strategy for all electronic databases is presented in Supplementary File S2.

### 2.3. Study Selection Process

Study selection consisted of two steps: first, screening of article titles and abstracts, and second, screening of full texts. Articles identified by our search strategy were exported to EndNote 21.2, and the first author removed duplicates.

The authors developed the inclusion and exclusion criteria (Table 1) applied throughout the selection procedure.

**Figure 1:**
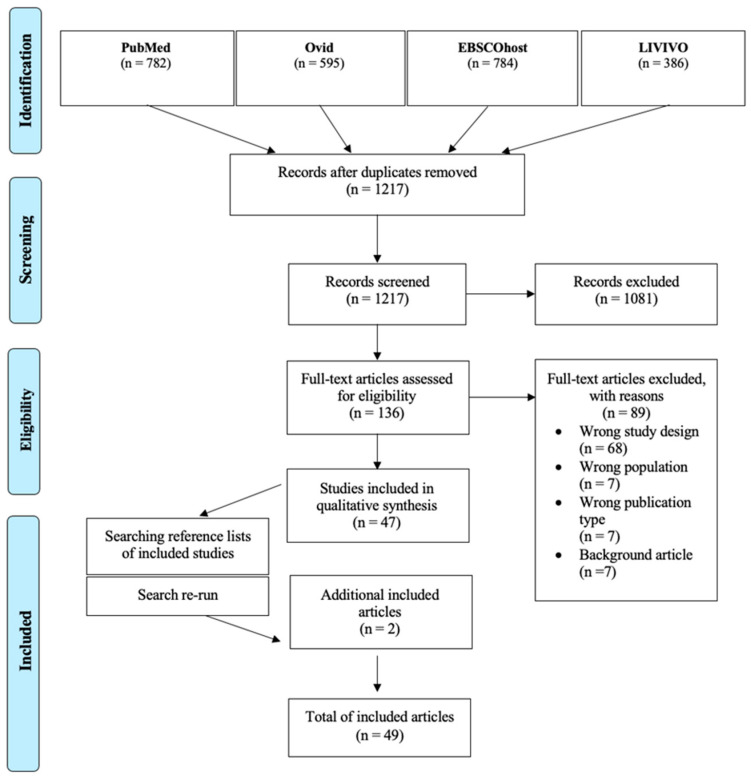
Study flow diagram, according to PRISMA extension for scoping reviews [18].

These criteria were used in both the first and second stages of screening. To determine eligibility for inclusion according to the agreed inclusion and exclusion criteria, the titles and abstracts of the articles were screened using the web-based software “Rayyan” (https://rayyan.ai/, last time accessed on 1 August 2023). The authors screened all titles and abstracts for inclusion and exclusion criteria (B.K., A.J.S.). H.O. confirmed the selection of articles. The reviewers then analyzed the full texts and assessed the inclusion criteria. The main reasons for the exclusion of an article were documented. The approach used to select studies that met the objectives of this scoping review is shown in Figure 1.

## 3. Results

### 3.1. Selection Process

The preliminary search yielded 1217 studies, of which 136 were deemed suitable for full-text screening. A total of 89 articles were excluded from further consideration due to the following reasons: 68 articles were deemed to have an inappropriate study design, 7 articles were excluded because they did not align with the specified population, 7 articles were excluded because they did not meet the criteria for the specified publication type, and 7 articles were excluded because they were background articles. A subsequent iteration of the search strategy yielded two additional articles, which were then included. Ultimately, 49 publications were deemed suitable for inclusion in the review.

### 3.2. Characteristics of the Studies

Forty-nine articles were published between 1980 and 2022, with 38% published between 2015 and 2020 (Table 2). Most studies were conducted in North America (46%) and Europe (34%). Immunofluorescence was the primary method of data collection (53%). Most of the studies were published by researchers associated with the departments of otolaryngology (65%) at various institutions.

### 3.3. Type and Distribution of Immune Cells in Rodent Ear

Twenty-five studies were dedicated to analyzing the inner ear of mice (Table 3), whereas five studies inspected the inner ear of guinea pigs (Table 4). Additionally, four studies examined the inner ear of rats and other species (Table 5). Together, these studies proved that the healthy, intact inner ear contains a population of resident macrophages (Table 3, Table 4 and Table 5).

Takahashi et al. (1988) demonstrated that the murine cochlea and the endolymphatic sac (ES) harbor macrophages [19]. In the guinea pig cochlea, macrophages were found in the lumen and perisaccular region of the ES [20]. Lang et al. (2006) provided evidence of hematopoietic cells’ ability to enter the cochlea and remain in both the modiolus and spiral ligament [21].

Various surface markers characteristic of monocytes and macrophages (Appendix A) have been used in animal studies and have shown that cochlear macrophages express Iba1, F4/80, CD68, CD163, and CX3CR1 on their surface (Table 3, Table 4 and Table 5). Iba1 (ionized calcium-binding adapter molecule 1) is a calcium-binding protein involved in macrophage activation, phagocytosis, and motility [22,23]. Okano et al. (2008) transplanted hematopoietic stem cells expressing green fluorescent protein (EGFP) into previously irradiated C57BL/6 mice [24]. Six months after transplantation, EGFP-positive cells were observed in the inner ear, most expressing Iba1 on their surface. The Iba1^+^ cells were observed in the spiral ligament, close to the spiral ganglia, and proximal to the basilar and Reissner’s membranes. Wakabayashi et al. (2010) demonstrated the presence of Iba1^+^ cells in the murine cochlea’s apical, medial, and basal turn [25].

Another well-characterized rodent macrophage marker expressed at high densities on the surface of mature macrophages is the cell surface protein F4/80 [26,27]. In addition to Iba1^+^ cells, F4/80 expressing cells were also observed in the spiral ligament and spiral ganglion [24]. The fractalkine receptor (CX3CR1) is a transmembrane glycoprotein and chemokine involved in the adhesion and migration of immune cells [15]. Few resident CX3CR1^+^ macrophages were observed in the osseous part of the spiral lamina [28].

The leukocyte common antigen (LCA), also known as cluster of differentiation-45 antigen (CD45), is a membrane-bound glycoprotein with tyrosine phosphatase activity that is expressed by all leukocytes [29]. The CD45^+^ cells were found in the spiral ligament, scala tympani, and spiral lamina, near the spiral ganglion cells, and under the basilar membrane (Table 3, Table 4 and Table 5). CD45^+^ cells were also observed to be present in the spiral limbus, the scala tympani, and the scala vestibuli, and their number increased significantly after acoustic overstimulation. Supporting these observations, CD45^+^ cells were reported to be distributed along the entire length of the basilar membrane [30].

Three studies have identified the presence of mast cells in rodents’ inner ears, specifically in the modiolus, spiral limbus, and ES [30]. To visualize the mast cells, the glycoprotein avidin, which has a high affinity for heparin in the mast cell granule, was employed as a staining agent, as this method has been established for use with mast cell granules [31].

**Table 3:** Types and distribution of immune cells in the inner ear of the mouse.

Number of Specimens Analyzed	Identified Immune Cell(s)	Antibodies Used for Identification	Immune Cell Distribution	Quantification	Refs.
4–5	Macrophages	IL-1b, Arg1, F4/80	lateral wall, OC, spiral ganglion, cochlear nerve,	-	[32]
3–7	Macrophages	Iba1, CD11b, CD68, F4/80	osseous spiral lamina, Rosenthal’s canal, lateral wall	-	[33]
-	Macrophages	CD45, F4/80, Iba1, Ly6C	basilar membrane, OC, spiral ligament, spiral limbus, neural region in the osseous spiral lamina, ganglion neurons and modiolus. CD45^+^ cells: scala tympani side of mesothelial cells, OC, along the spiral vessels, spiral ligament, spiral limbus, neural regions of the cochlea	CD45^+^ cells: average number of cells per 1 mm length of the basilar membrane decreased from 35.4 ± 6.4 during the P1–4 to 26.2 ± 6.5 at P10 and to 16.8 ± 2.4 during P17–21; CD45^+^ cells in the apical region: 31.9 ± 11.7; CD45-positive cells in the middle region: 24.1 ± 8.2; CD45^+^ cells basal: 58.3 ± 2.7; spiral limbus: 51.3 ± 3.2 cells/0.1 mm^2^ at P4 to 23.8 ± 3.2 at P10 and to 15.6 ± 1.3 at P17; spiral ganglion region: 41.7 ± 3.2 cells per 0.1 mm^2^ at age P4, 35.3 ± 3.3 at age P10, 27.3 ± 1.2 at P17,	[34]
-	Macrophages	CD45, F4/80	across the entire length of the sensory epithelium	average number of macrophages increased from 25 ± 6 at the age of 1 month to 29 ± 3 at the age of 3–5 months and 30 ± 6 at the age of 10–12 months. basal section: average number of macrophages was reduced from 49 ± 15 for the young group to 43 ± 11 for intermediate-aged group, and further down to 22 ± 8 for the 10–12-month group	[35]
5	Macrophages	CD45, Iba1, Ly6C	spiral lamina, basilar membrane, scala tympani, osseous spiral lamina, among the peripheral nerve bundles of ganglion neurons	spiral lamina: 30 ± 5 basal membrane: 114 ± 11	[36]
-	Macrophages	-	in the lumen of the ES	-	[37]
8	Immune cells	MHC class II	lateral wall, OC, modiolus, spiral ganglion	-	[38]
13	Macrophages	BrdU, CD68, CD3, CD45	spiral ligament, stria vascularis	5–7 inflammatory cells per 30 μm section	[15]
5	Macrophages	GFP	OC	-	[39]
4	Macrophages	GFP	sensory epithelium of utricles	1.4 ± 0.6 macrophages/100,000 μm^2^	[40]
-	Macrophages	Iba1, CD11b, F4/80, CD68	IBA1-positive macrophages: spiral ganglion, spiral ligament, stria vascularis, intraluminal surface of perilymphatic space	IBA1-positive macrophages: 105 ± 50.8 at P0, 411 ± 36.6 at P3, 492 ± 49.9 at P6, and 513 ± 17.8 at P21 in the spiral ganglion (/mm^2^, mean ± SEM); 628 ± 61.9 at P0, 666 ± 47.3 at P3, 438 ± 58.8 at P6, and 270 ± 37.3 at P21 in the spiral ligament; and 0 ± 0 at P0, 430 ± 41.5 at P3, 583 ± 96 at P6, and 356 ± 32 in the stria vascularis	[41]
10	Leukocytes (Monocytes, Lymphocytes, Neutrophils)	CD45, CD54	scala vestibuli, scala media, scala tympani, spiral ligament, stria vascularis, modiolus, and limbus	only a few labeled cells	[42]
-	Perivascular resident macrophage-like melanocyte	F4/80	capillaries of the stria vascularis at the apical, middle, and basal turns	1-month-old animals: 352 ± 39/mm^2^ stria area 21 months: 247 ± 35/mm^2^ stria area	[43]
12	Macrophages	Iba1	spiral ganglion, spiral ligament, stria vascularis	spiral ganglion: 3.42 ± 0.29, spiral ligament: 4.35 ± 0.36, stria vascularis: 6.83 ± 0.69	[44]
5	Macrophages	CD45, Iba1, CD68, F4/80	spiral ganglion, spiral ligament	-	[24]
40	Perivascular resident macrophages	F4/80, CD68, CD11b, MOMA2	along capillaries of the blood–labyrinth barrier in the stria vascularis	-	[45]
40	Perivascular resident macrophages	F4/80	between marginal and basal layers of stria vascularis	-	[46]
5	Mac-1-, Lyt-1-, and Lyt-2-positive cells and Immunoglobulin-positive cells	Mac-1, Lyt-1, Lyt-2, immunoglobulins classes M, G and A	cochlea: no positive cells found; ES: Lyt-1+ and IgM+ cell in perisaccular region, Mac-1 and IgG-positive cells in the ES region	-	[19]
-	T cells, B cells (IgM-, IgG- and IgA-positive), Macrophages	Thy-1, Lyt-1, Lyt-2, immunoglobulins classes M, G and A	Thy-1’ cells: throughout the ES Lyt-l+ cells: throughout the ES Mac-1’ cells: lumen ES IgM+ cells: subepithelial region IgG’ cells: lumen and perisaccular space	-	[47]
6	Lymphocyte	CD4, CD8a,	ES	-	[48]
4	Macrophages	CD45, F4/80, MHCII and Tlr4	lateral wall, basilar membrane, scala tympani	~1 cell/100 µm	[49]
3	Macrophages	CD45, Iba1	spiral ganglion, spiral ligament	less than one cell per 10,000 µm^2^	[25]
7	Macrophages	CD4, CD11c, CD14, CD45, CIITA, F4/80 and MHCII	basilar membrane	95.4 ± 16.9	[30]
5	Perivascular resident macrophage-like melanocytes	F4/80, GSTα4	semicircular canal ampullae, utricle, saccule, and semicircular canal	utricle: 225 ± 43/mm^2^; saccule 191 ± 25/mm^2^; horizontal ampullae 212 ± 36/mm^2^; anterior ampullae 238 ± 36/mm^2^; and posterior ampullae 223 ± 64/mm^2^	[50]
8	Macrophages	-	spiral ligaments, stria vascularis, Reissner’s membrane	-	[51]

**Table 4:** Type and distribution of the immune cells in the inner ear of the guinea pig.

Number of Specimens Analyzed	Identified Immune Cell(s)	Immune Cell Distribution	Quantification	Refs.
22	Leukocytes	Around the vein.	-	[52]
5	Mast cells	Surrounding capillaries in the subepithelial connective tissue of the ES, but not detected in the stria vascularis. Connective tissue of the ES but not in other parts of the inner ear.	-	[53]
-	Lymphoid cells, Macrophages, Lymphocytes, Plasma cells, Mast cells	ES.	-	[54]
-	Macrophages, Lymphocytes, Plasma cells, Mast cells	Perisaccular blood vessels, ES.	-	[55]
- (Guinea pigs, humans)	Mast cells	In the subepithelial connective tissue of the ES. In no other part of the inner ear.	500–700 per sac, average of 605	[56]

All studies used eosin–hematoxylin staining to identify the cells.

**Table 5:** Types and distribution of immune cells in the inner ear of the rat and monkey.

Number of Specimens Analyzed	Identified Immune Cell(s)	Antibodies Used for Identification	Immune Cell Distribution	Quantification	Refs.
18 (rats)	Leukocytes, Macrophages	CD45, ED1, CD68	spiral ganglion, OC	-	[57]
- (rats)	Macrophages/Microglia	Iba1	cochlear aqueduct, stria vascularis, spiral ligament, endolympathic duct	-	[58]
- (rats, mice)	Mast cells	c-Kit/CD117, MC chymase, MC tryptase	modiolus, the spiral limbus of both species, Reissner’s, no MCs were detected in or close to the OC	Wistar rats: P1 average 17 ± 12.3, P3 average 14.7 ± 8.6, P5 average 9.8 ± 6.2, P7 average 4.7 ± 3.5, and P9 average 2.6 ± 2.1	[59]
6 (monkeys)	Macrophages, Plasma cells, Lymphocytes	-	the rim of the round window membrane	-	[60]

### 3.4. The Type and Distribution of Immune Cells in the Human Inner Ear

Sixteen of the included studies analyzed the human inner ear. The specimens utilized in human research were collected from patients undergoing surgical removal of the cochlea due to meningioma or other diseases that did not affect the cochlea, as well as from deceased individuals without a known history of hearing or balance disorders. In two instances, post-mortem temporal bones of patients who had undergone unilateral cochlear implantation were examined, with the inclusion of specimens from the non-implanted ear [61,62]. The data obtained from the latter were incorporated into the present study. In the included studies, the immunohistochemical stainings were performed with the antibodies against CD163, Iba1, and CD68 (see Table 6 and Appendix A). The CD163 protein, expressed exclusively by monocytes and macrophages, acts as a scavenger receptor [63]. CD163^+^ cells were located in various areas in the human inner ear, including the spiral ligament, adjacent to the basilar and Reissner’s membranes, in the spiral lamina and spiral limbus, along the blood vessels, close to the spiral ganglion neurons, in the vestibular organ, and in the ES. CD68, similarly to CD168, is a scavenger receptor exclusively expressed by monocytes and macrophages and belongs to the group of lysosomal/endosomal-associated membrane glycoproteins [63]. CD68^+^ cells have been identified in the human inner ear, located near the basilar and Reissner’s membranes near spiral ganglion neurons and the ES. Iba1^+^ cells were detected in various regions of the inner ear, including the spiral ligament, in proximity to the basilar and Reissner’s membranes, in the spiral lamina and limbus, alongside blood vessels, in the vestibular organ, proximal to spiral ganglion neurons, and in the ES. This distribution pattern is consistent with that of CD163 and CD68. Notably, macrophages in the human inner ear exhibit more significant expression of Iba1 than CD163 and CD68. Furthermore, Iba1^+^ macrophages were detected within the lumen of the perisaccular blood vessels of subepithelial tissue and the proximal section of the ES [54,63,64].

Additionally, immunohistochemical investigations illustrated the presence of lymphocytes (T, B, and Langerhans cells), leukocytes, and mast cells in the ESs of human inner ears (Table 6).

### 3.5. Summary of Immune Cell Types and Distribution across Species

**Figure 2:**
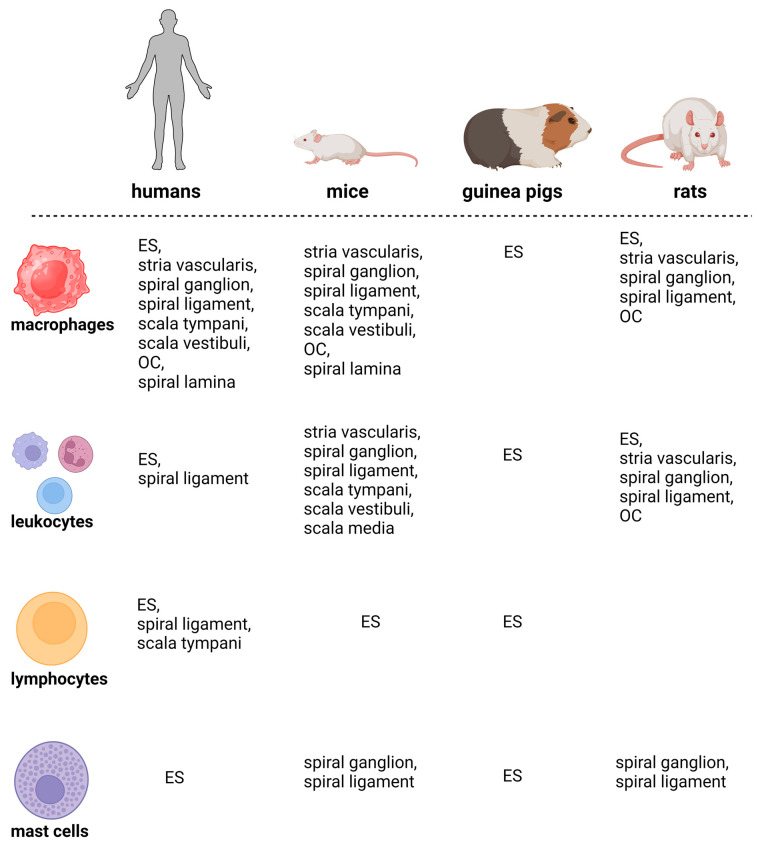
Schematic presentation of the main findings of this scoping review. ES, endolymphatic sac; OC, organ of Corti. Created with BioRender.com.

Several studies have shown that immune cells have been detected at several sites in the mammalian inner ear (Table 3, Table 4, Table 5 and Table 6, Figure 2). Macrophages were observed in the ES, stria vascularis, spiral ganglion, spiral ligament, scala tympani, scala vestibuli, OC, and spiral lamina. Leukocytes and lymphocytes were found in the ES, spiral ligament, and lateral portion of the cochlea. Mast cells were observed in the ES, modiolus, and spiral ligament.

## 4. Discussion

**Figure 3:**
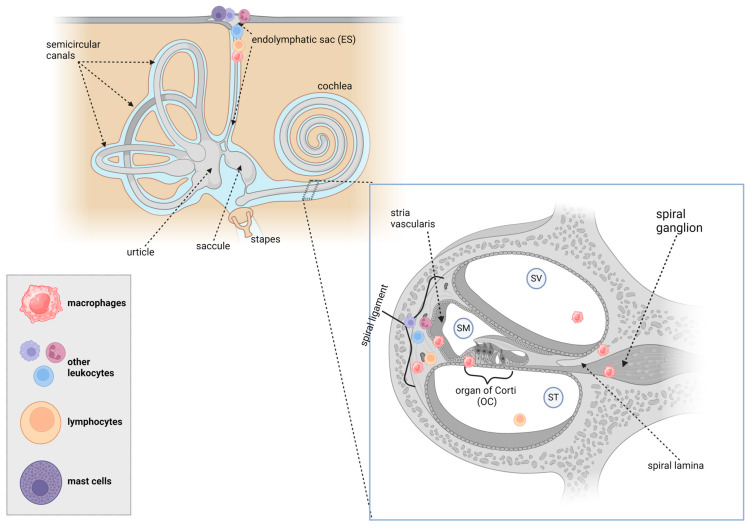
The location of macrophages, leukocytes, lymphocytes, and mast cells at a steady state in the human inner ear with emphasis on the cochlea. ST, scala tympani, SM, scala media, SV, scala vestibuli. Created with BioRender.com.

This scoping review assesses the current literature on the types and distribution of immune cells in the mammalian inner ear under steady-state conditions, with a particular focus on control, experimentally unmanipulated animals. The identification of immune system cells was based on the expression of different surface molecules, including CD45, CD4, CD8, F4/80, Iba1, CD68, and CD163. Immunocytes were observed in various structures within the inner ear, as detailed in Table 6 and Figure 3. The findings suggest that innate and acquired immune cells are integral components of the mammalian inner ear under normal physiological conditions. Further research in inner ear biology is needed to understand the functions of the resident immune cells, which is of utmost importance and should be a priority for the scientific community.

In support of these observations, Jean et al. recently published the results of single-cell transcriptomics performed on mouse cochleae obtained from animals at postnatal days 8, 12, and 20 [75]. According to these data, various innate and adaptive immune cells (neutrophils, B cells, T cells, NK cells, monocytes, macrophages, and mast cells) are present in the cochlea. However, due to the methodology used, it is unclear which cells are located in the solid cochlear tissue and which are located in the cochlear blood vessels (which were not separated from the rest of the cochlear tissue during preparation). However, the presence of macrophages, which, unlike monocytes, do not circulate in the blood [76], suggests that tissue-resident immune cells were also included in this analysis.

It has been documented that resident leukocytes can be found in several human and animal body tissues. They maintain local homeostasis, patrol tissues searching for pathogens or toxins, repair potential cell damage, and remove dead or damaged cells [76,77,78]. Notably, while they retain the typical properties of a particular type of immune cell, they also possess specific characteristics unique to the particular tissue in which they reside. To illustrate, resident lung macrophages play a role in maintaining pulmonary homeostasis through surfactant uptake [79]. Furthermore, they inhibit the initiation of inflammatory responses to innocuous particulate matter [80]. The resident cardiac macrophages, originating from the yolk sac and fetal liver progenitors, express a range of growth factors and matrix metalloproteinases that facilitate cardiac tissue remodeling. Moreover, macrophages in the atrioventricular node are involved in myocyte repolarization and electrical conduction [81]. The homeostatic function of other immunocytes has yet to be the subject of extensive investigation, representing a significant avenue for further research in inner ear biology.

The particular contributions of resident immune cells to inner ear homeostasis can be elucidated by analyzing auditory function in mutants. Unfortunately, only a limited number of such analyses have been conducted. One such work demonstrates that SCID mice, which have a mutation in the Prkdc (scid) gene responsible for double-stranded DNA repair and, thus, do not produce functional T and B cells, have normal hearing abilities [82]. Interestingly, the same study showed that transferring bone marrow cells purged from T cells from MRL/Mp-lpr/lpr mice known to have systemic autoimmunity associated with systemic lupus erythematosus to SCID animals increased hearing thresholds, suggesting the detrimental role of B cells in that process. The hearing abilities of NUDE mice, which do not develop T cells, Rag1 mice, which lack T and B cells, and other immunodeficient animal models remain unknown. Further interdisciplinary research following the principles of the 3Rs and 4Rs of research would be required to gain a deeper understanding of the subject matter.

An alternative approach to gaining information about the resident immune cells is to observe otologic pathologies in patients with immunodeficiencies to elucidate the specific contributions of distinct immune system cell types to inner ear pathologies. Clinical evidence supports the association between primary immunoglobulin immunodeficiencies and hearing loss [83,84]. However, the etiology of hearing loss appears to be mixed in these disorders and often involves infections of the middle ear. The prevalence of hearing loss in persons with secondary immunodeficiencies, such as those infected with HIV or AIDS, is relatively high. A review paper analyzing hearing loss in HIV-infected children estimated it to be between 6% and 84% based on the data analyzed [85], with conductive hearing loss occurring more often than sensorineural or mixed hearing loss. The authors explain the wide range of results by the heterogeneity of the sampling and the audiometric methods used. A study by van der Westhuizen and colleagues (2013) found that among adults living with HIV, the prevalence of hearing impairment was 27.5%, tinnitus was 26%, and dizziness was 25% [86]. Again, mixed etiologies, including antiviral medicine ototoxicity, have been proposed to explain the otologic symptoms. Still, one cannot exclude the possibility that a virus-depleted number of CD4+ cells contributes to the phenotype. A study by Iwai et al. (2021) demonstrated a correlation between a declining fraction of circulating CD4+ lymphocytes and accelerated age-related hearing loss in mice [87]. The authors attributed the effect to the systemic impact of CD4+ depletion and did not observe the presence of mononuclear cells in the cochleae. Nevertheless, as these cells are usually present in small numbers, they were not detected by the hematoxylin and eosin staining used in that paper.

At present, the population of resident immune cells with the most significant amount of comprehensive understanding is that of macrophages. Nevertheless, the current evidence regarding the impact of resident macrophages on inner ear structures remains inconclusive. On the one hand, resident macrophages positively influence hair cell loss rates, tissue repair, and removal of cellular debris [28,88]. Notably, the development of ribbon synapses during the onset of hearing requires resident macrophages [89]. Additionally, macrophages are required to repair noise-damaged inner hair cell synapses [90]. On the other hand, the ability of macrophages to produce cytotoxic substances such as reactive oxygen species (ROS) and to initiate inflammatory responses after acoustic trauma suggests their potential contribution to hair cell loss [15]. The properties of resident cochlear macrophages were recently summarized in a review by Hough et al. [91]. The authors concluded that macrophages contribute to cochlear homeostasis under normal physiological conditions. However, in some cases, they can be primed and activated, which might lead to cochlear damage, resulting in hearing loss.

The pathologies of the inner ear with the potential involvement of the resident immune cells include autoimmune inner ear disease (AIED) and noise-induced hearing loss. Treating AIED and noise-induced hearing loss necessitates targeting the inflammatory response and proinflammatory cytokines [92]. McCabe et al. (1979) demonstrated that the administration of glucocorticoids to patients with AIED resulted in an improvement in hearing loss [93]. Corticosteroids remain a mainstay of first-line therapy for AIED [94]. Furthermore, clinical trials have shown that corticosteroids, including dexamethasone and methylprednisolone, effectively prevent noise-induced hearing loss [92]. Inhibition of inflammatory mediators, such as tumor necrosis factor-α (TNF-α) or interleukin-1 (IL-1), represents another potential treatment strategy for AIED. For example, a clinical study demonstrated the efficacy of the TNF-α inhibitor infliximab when administered intratympanically in treating hearing loss in patients with AIED [95,96]. However, a review of the efficacy of anti-inflammatory biologics used to treat off-label AIED revealed inconsistencies in the clinical outcomes. These inconsistencies were attributed to sample size and composition variations, biologics, and other drugs applied simultaneously. This highlights the necessity for more rigorous studies to gain a deeper understanding of the efficacy of these biologics in treating off-label AIED [97].

### 4.1. Limitations

The analyzed studies exhibited notable heterogeneity. Variation was observed not only in methodology but also in test samples. In some studies, the number of immune cells was not determined, but their presence was noted. It is also important to note that some relevant studies may have been overlooked despite a comprehensive search strategy.

### 4.2. Future Directions and Conclusions

In mammals, the inner ear contains a resident population of immune cells that maintain a steady state under normal conditions. The precise manner in which the resident immune cells contribute to inner ear homeostasis and their potential role in the pathogenesis of inner ear diseases remains unresolved and requires further investigation. Moreover, the function of the resident immune cells within the inner ear remains unclear. A more detailed and systematic examination of the spiral ligament, stria vascularis, and the modiolar spiral vein in the context of immune cells may facilitate a comprehensive understanding of inner ear immunophysiology and pathology and develop innovative therapeutic interventions. Furthermore, no study distinguished between resident and infiltrated immune cell populations using the same tissue sample, which presents an intriguing opportunity for further investigation. This fascinating interdisciplinary field necessitates close collaboration between otologists, inner ear biologists, and immunologists.

## 5. Conclusions

This review examines the current knowledge regarding resident immune cells in the inner ear of various mammalian species. A comprehensive analysis of the findings revealed the presence of resident immune cells in the inner ear of all organisms studied under normal physiological conditions. The knowledge in this review may facilitate future research into the physiology and diseases of the inner ear. However, further research is needed to clarify the role of resident immune cells in the inner ear.

## Figures and Tables

**Table 1:** Inclusion and exclusion criteria used to select manuscripts.

**Inclusion Criteria**	Peer-reviewed, full-text articles reporting original data; Papers primarily or partially focused on immune cells within the inner ear; Articles that primarily consider cochlear implants, sensorineural or noise-induced hearing loss were included only if a control group was represented; Research performed on the mammalian inner ear.
**Exclusion Criteria**	Conference abstracts, review papers, letters to the editor, opinion pieces, news, or case reports; Not primarily concerned with the inner ear (e.g., middle ear); Articles mainly dealing with systemic autoimmune diseases (e.g., granulomatosis with polyangiitis, spondylarthritis); Lack of at least one of the following terms in their title/abstract or the keywords in combination with immune cells: cochlea, inner ear, endolymphatic duct, endolymphatic sac, the organ of Corti (OC), vestibular organ, vestibular system; Articles were excluded when the study design used an intervention in the inner ear in the controls.

**Table 2:** Characteristics of the included studies (n = 49).

		Number of Studies (% of All Studies Included)
Year of publication	<1980	3 (6%)
	1980–1985	2 (4%)
	1985–1990	4 (8%)
	1990–2000	3 (6%)
	2000–2005	3 (6%)
	2005–2010	7 (14%)
	2010–2015	6 (12%)
	2015–2020	19 (38%)
	2020–2022	2 (4%)
Continent	Europe	17 (34%)
	Asia	9 (18%)
	North America	23 (46%)
Method used	Immunohistochemistry	16 (32%)
	Immunofluorescence	26 (53%)
	Electron microscopy	7 (14%)
Research profile of publishing group	Otorhinolaryngology	32 (65%)
	Pathology	2 (4%)
	Hearing research center	9 (18%)
	Multiple departments	2 (4%)
	Other	4 (8%)

**Table 6:** Types and distribution of immune cells in the human inner ear.

Number of Specimens Analyzed	Identified Immune Cell(s)	Antibodies Used for Identification	Immune Cell Distribution	Quantification	Refs.
60	T cells (CD4 and CD8), B cells, Langerhans cells, IgA- or IgG-containing lymphoid cells, Macrophages	T cells: CD45, CD8, CD4, CD6, CD5, DAKO-UCHL-1, DAKO-T1; Langerhans cells: CD1; B cells: IgA, IgG, CD22; macrophages: anti-lysozyme	T cells: epithelium, lumen, and stroma of ES; B cells: stroma and occasionally lumen of ES; Langerhans cells: occasionally epithelial layer of ES; IgA- or IgG-producing B cells: perisaccular area and lumen of ES macrophages; lumen and perisaccular stroma.	-	[65]
-	Plasma cells with cytoplasmic IgA	IgM, IgG, IgA	Perisaccular region of ES.	-	[66]
-	Macrophages, Lymphocytes	IBA1, CD68, CX3CL1, TLR4, CD11b, CD4, CD8a, MHCII	IBA1-positive macrophages: subepithelial tissue and epithelium of ES in the stria vascularis and spiral ganglion; spiral ligament. TLR4-positive cells: among subepithelial cells in the intermediate ES. CD4- and CD8-positive lymphocytes: in the ES.	-	[67]
6	Macrophages	IBA1	Lateral wall, including the spiral ligament, scala vestibule and scala tympani, spiral limbus, spiral lamina, spiral and vestibular ganglion; in the OC.	-	[68]
5	Macrophages, Lymphocytes	IBA1, CD4, CD8, CD11b, CD68, MHCII, CX3CL1	IBA1-positive macrophages: connective tissue and epithelium of the ES; stria vascularis, around the blood vessels; spiral ligament, scala vestibuli and tympani; spiral limbus, spiral lamina. In the OC, surrounding spiral ganglion. CD4- and CD8-positive lymphocytes: around modiolar blood vessels and along the border of Rosenthal’s canal; spiral ligament.	-	[69]
5	Macrophages, Lymphocytes	IBA1, MHCII, CX3CL1, CD11b, CD4, CD8α	CD4- and CD8-positive lymphocytes around vessels of the modiolus and along the border of the Rosenthal’s canal; scala tympani, spiral ligament. IBA1-positive macrophages: stria vascularis, among the neurons in the Rosenthal’s canal, OC.	-	[69]
5	Macrophages, Lymphocytes	IBA1, MHCII, CX3CL1, CD11b, CD4, CD8α	CD4- and CD8-positive lymphocytes: around vessels of the modiolus, spiral ligament. IBA1-positive macrophages: stria vascularis, among the neurons in the Rosenthal’s canal, in the modiolus.	-	[70]
5	Macrophages	IBA1, CD163	IBA1-positive macrophages: stria vascularis, in the osseous spiral lamina and Rosenthal’s canal. CD163-positive macrophages: spiral ligament, osseous spiral lamina, and Rosenthal’s canal.	Apical turn: 2.7 ± 1.0; middle turn: 7.8 ± 3.5; basal turn 11.3 ± 6.5. Spiral ligament apical turn: 1.0 ± 1; middle turn: 4.0 ± 3.3; basal turn: 4.9 ± 3.0.	[71]
5	Macrophages, Lymphocytes	IBA1, CD11b, CD4, CD8a, TLR4, MHCII	IBA1-positive macrophages: subepithelial tissue, epithelium, perisaccular connective tissue, sac lumen; stria vascularis, spiral ganglion, spiral ligament; CD4- and CD8-positive lymphocytes: in the ES.	-	[72]
114	Macrophages	CD163, CD68, Iba1	CD68-, IBA1-, and CD163-positive macrophages: spiral ligament, along the basilar membrane on the perilymphatic compartment, within the tunnel of Corti, along Reissner’s membrane, in the osseous spiral lamina, along blood vessels, among the spiral ganglion cells, in the endolymphatic duct.	-	[63]
80	Macrophages	IBA1	Crista ampularis, neuroepithelium, subepithelial stroma, mid-stroma.	-	[61]
20	Macrophages	Iba1	OC, under the basilar membrane, in the perilymphatic compartment, around the blood vessels; stria vascularis, osseous spiral lamina.	-	[62]
-	Plasma cells, Monocytes, and/or Macrophages	-	Within the epithelium, around the ES.	-	[73]
10	Monocytes, Macrophages, Granulocytes, Leukocytes	Leu4, Leu3, Leu2, Bl, T29/33, OKMl	Macrophages: lumen of the human ES. Monocytes: limited to the ES vasculature. Leukocytes: within the subepithelial space. Lymphocytes: within the subepithelial space.	-	[64]
-	Macrophages, Leukocytes	IBA1, CX3CL1, CX3CR1, P2Y12, MHCII, CD11b, CD117, CD19, CD8α, CD4, CD68, TLR4, TMEM119, vimentin, collagen type IV	IBA1-positive macrophages: lateral cochlear wall, spiral limbus, Reissner’s membrane, osseous spiral lamina, surrounding blood vessels, the wall of the scala vestibuli and tympani, a few occasionally in the OC, around the spiral ganglion. Macrophages: spiral ligament among types II, IV, and V fibrocytes, spiral ganglion. CD4- and CD8-positive lymphocytes: modiolus around blood vessels.	-	[74]
2	Macrophages	osmic acid- and iron–hematoxylin-stained	The connective tissue of the planum semilunatum in the canalicular wall, surrounding a big vessel of the stria vascularis.	-	[67]

## Data Availability

No new data were created in this work.

## Supplementary Materials

The following supporting information can be downloaded at https://www.mdpi.com/article/10.3390/cells13181528/s1, File S1: protocol; File S2: search strategy. References [1,2,9,14,15,16,29,46,57,98,99,100,101,102] are cited in the supplementary materials.

## Appendix A

**Table A1:** A comprehensive list of the immune markers employed in the publications included in this review. The table also includes the alternative names of the molecules, their ligands, and their principal functions. The data regarding the cell type in which the markers are expressed (T cells, B cells, NK cells, macrophages, granulocytes, and mast cells) pertain to humans and mice.

Marker	Alternative Name	Ligand	T Cell	B Cell	NK Cell	Macrophage/Monocyte	Granulocyte	Mast Cell	Function	References
CD1	Leu6	Lipids and glycolipids		+		+			Lipid and glycolipid antigen presentation.	[103]
CD3	Leu4	TCR complex	+		+				Signal transduction	[103]
CD4	L3T4, Leu3a	MHC class II, IL-16, receptor for HIV	+		+	+			Signal transduction, receptor/coreceptor.	[103]
CD5	Lyt-1	CD72	+	+					Adhesion, regulation of T–B lymphocyte interaction.	[103]
CD6	T12	CD166	+						Activation/costimulation, adhesion, differentiation/development.	[103]
CD8	Leu2, Lyt-2	MHC class I	+						Signal transduction, receptor/coreceptor for MHC class I molecules.	[103]
CD117	c-KIT	Stem cell factor (c-kit ligand)						+	Pivotal role in the proliferation and differentiation of various cell types. Indispensable for maintaining hematopoietic stem cells in the bone marrow and mast cells’ development and function.	[103]
CD11b	Integrin αM, Ly-40	CD54, fibronectin	+	+	+	+	+		Adhesion, chemotaxis, apoptosis.	[103]
CD14	LPS Receptor	LPS				+	+		Pathogen recognition, inflammation.	[103]
CD22	SIGLEC-2	Sialic acid		+					B cell adhesion, immunoregulation, receptor/coreceptor, signal transduction.	[103]
CD45	Leukocyte common antigen (LCA)	CD150, galectin-1, CD2, CD3, CD4	+	+	+	+	+	+	T and B cell antigen receptor signaling, regulator of cell growth and differentiation.	[103]
CD54	ICAM-1, Ly-47		+	+		+		+	Cell adhesion, lymphocyte activation and migration.	[103]
CD68	LAMP4	Low-density lipoprotein, phosphatidylserine, apoptotic cells				+			Phagocytosis.	[103,104]
CD90	Thy-1	Not yet identified	+		+	+			Signal transduction, activation/costimulation, adhesion, differentiation/development.	[103]
CD163	SCARI1	Hemoglobin–haptoglobin complex				+			Scavenger receptor.	[105]
CD284	Toll-like receptor 4 (TLR4)	Lipopolysaccharide (LPS), high mobility group box 1 (HMGB1), heat shock proteins	+	+	+	+	+	+	Recognition of damage or pathogen-associated molecular patterns.	[106,107,108,109]
CX3CR1	Fractalkine receptor or G-protein coupled receptor 13	Neurotactin/fractalkine	+	+	+	+	+	+	Cell adhesion, migration, survival, proliferation, neuroprotection.	[110,111]
F4/80	EGF-like module-containing mucin-like hormone receptor-like 1	Not yet identified				+	+		immune response modulation and inflammation.	[112]
Iba1	Allograft inflammatory factor 1 (AIF-1)	Fimbrin				+			Membrane ruffling and phagocytosis.	[113,114]
Mac-1	Complement receptor 3	ICAM1, ICAM2	+	+	+	+	+		Phagocytosis, pattern recognition receptor.	[103]
