# Supplementary material for: Mammalian Inner Ear-Resident Immune Cells—A Scoping Review
Source: Cells. 2024 Sep 12;13(18):1528. doi: 10.3390/cells13181528 (PMC11430779; doi:10.3390/cells13181528)
Supplement: Supplementary file 1 [file cells-13-01528-s001.zip › Supplementary File S1.pdf]

Scoping Review Protocol

Immune cells in the mammalian inner ear under steady-state conditions – a scoping review

Betül Karayay, Heidi Olze, Agnieszka J. Szczepek

Department of Otorhinolaryngology, Head and Neck Surgery, Charité – Universitätsmedizin Berlin, Humboldt-Universität zu Berlin, and Berlin Institute of Health, Berlin, Germany

Abstract / Structured summary

Background: The inner ear has long been considered an immune-privileged organ. However, several studies have demonstrated that the healthy inner ear contains resident immune cells.

Objective: The present study undertakes a scoping review of the current state of research regarding the characterization and localization of immune cells in a steady state in the mammalian inner ear.

Design: Online databases (Pubmed, Medline (Ovid), CINAHL (EBSCO), Embase Classic+Embase 1947–2020 (Ovid) and LIVIVO) were used to identify papers with no year restriction. Systematic review articles, experimental studies, and clinical data in English and German were included. The search identified 49 relevant articles that were published between 1979 and 2022. The data were extracted and analyzed.

Results: The results demonstrated that resident immune cells were observed in various inner ear structures. In addition, several studies reported that ototoxic drugs, acoustic trauma, and infection caused a quantitative increase in the immune cells in the inner ear.

Conclusions: The healthy inner ear has resident immune cells. However, the answer to the question about their function remains elusive, opening a new avenue for inner ear biology research.

Introduction

The inner ear is a complex organ that enables hearing and balance[1,2,98]. When compared to the cell biology, biophysics, and genetics of the inner ear, the inner ear immunology received little attention [99]. The opinion was that the blood-labyrinth barrier, consisting of tight junctions in the capillaries of the stria vascularis (SV), intercepts immune cells, antigens, and antibodies [9,100,101]. However, earlier electron microscopy studies demonstrated the presence of macrophages in the noise-exposed cochlea of guinea pigs [14]. This discovery was followed by other reports about the existence of resident macrophages in the cochlea, as well as a small resident population of CD45+ immune cells in the lower section of the spiral ligament and spiral wall [15,16,29,46,102]. However, it is unclear what kind of information is available in the literature about the resident immune cells in the inner ear under steady-state conditions, what potential role these cells have in the immune response in the inner ear, and in which structures of the inner ear can be found. Therefore, a scoping review was chosen to analyze and describe the research done in this area [57] as well as to identify any gaps in existing literature.

Aim of the review

Aim of this review is to scope the literature describing resident immune cells in the inner ear of different species under steady state conditions and provide an overview of these immune cells.

Research questions

1) What type of immune cells are described in the mammalian inner ear under steady-state conditions?

2) What is the distribution of immune cells in the inner ear?

3) What is the potential role of the resident immune cells in the inner ear?

Methods

Protocol

To draft our protocol and scoping review, we will follow the methods described by Arksey and O'Malley and the Preferred Reporting Items for Systematic Reviews and Meta-Analyses extension for Scoping Reviews (PRISMA-ScR) guidelines for reporting scoping reviews.

Information sources

We will search online databases such as Pubmed, Medline (Ovid), CINAHL (EBSCO) and LIVIVO. Each database will be searched with matching search terms (S2_file). The search will include free text-words and MeSH terms as well as Boolean operators. We will record our search including information such as: each database; dates of the searches; applied limits or filters; the number of records found (for each database) and search terms from at least one electronic database. Our search will have a language (English and German) and no year restriction. The reference list of papers which meet our including criteria will be screened for missing papers.

Eligibility criteria:

- Study type: We will consider only full-text papers that report original data. Conference abstracts, review papers, letters to the editor, and opinion pieces, news, or case reports will be excluded.
- Inclusion criteria:
  - o To be included in the scoping review, papers needed to deal with immune cells in the inner ear.
  - o Papers who mainly deal with noise induced hearing loss or cochlear implants will be included if they show a control group without any conditions.
- Exclusion criteria:
  - o We will exclude paper when they mainly deal with systemic autoimmune diseases, such as granulomatosis with polyangiitis or spondyloarthritis

o We will exclude papers if they not primarily concerned with the inner ear (e.g. middle ear).

o Lack of at least one of the following terms in the title or the keywords in combination with immune cells: cochlea, the organ of Corti, inner ear will be excluded

Study selection

• First, we will import the papers into EndNote and remove the duplicates. To determine eligibility for inclusion against the agreed inclusion and exclusion criteria the titles and abstracts of the papers will be the screened with the screening software “Rayyan”.

o We will remove the articles, which were not meeting the eligibility criteria.

o The screening of the data will be done by one reviewer and verified by a second reviewer.

• Secondly, we will study all potentially relevant publications as full text and fulfillment of the inclusion criteria will be evaluated.

o The main reasons why papers are excluded will be documented.

o We will present an adapted PRISMA flow diagram to document the number of papers identified, screened, assessed for eligibility, and included or excluded.

Data extraction

We will create an Excel table to facilitate the systematic evaluation of the studies and identify relevant variables. Data items will include:

• Article identifiers (first author, year of publication, title)

• Country national or regional

• Article type;

• Study characteristics (sample size, design, population, inclusion, and exclusion criteria)

• Setting (species, age)

• Data collection method

• Institutional affiliation/research department

In another Excel table, the following data will be collected:

• Species

• Cell type

• Cell distribution

• Reference number

Data synthesis

First, the descriptive findings of the literature regarding the number of articles published by date and the institutional affiliation of the first author will be introduced. The results will be presented in a tabular form. The tables will show an overview of the included studies and the reported immune cells in healthy inner ears sorted by species and their distribution.
