# Supplementary material for: Mammalian Inner Ear-Resident Immune Cells—A Scoping Review
Source: Cells. 2024 Sep 12;13(18):1528. doi: 10.3390/cells13181528 (PMC11430779; doi:10.3390/cells13181528)
Supplement: Supplementary file 1 [file cells-13-01528-s001.zip › Supplementary File S2.pdf]

Search Strategy for each database:

Database: PubMed/Medline Search Strategy (Literature Search performed: August 22, 2022)

1. "Cochlea"[MeSH Terms] OR "cochlea/cytology"[MeSH Terms] OR "cochlea/immunology"[MeSH Terms] OR "ear, inner"[MeSH Terms] (53657)

2. "antibody-producing cells"[MeSH Terms] OR "antigen-presenting cells"[MeSH Terms] OR "Leukocytes"[MeSH Terms] OR "Bone Marrow Cells"[MeSH Terms] OR "Macrophages"[MeSH Terms] OR "Mast Cells"[MeSH Terms] OR "Myeloid Cells"[MeSH Terms] OR "Phagocytes"[MeSH Terms] (1009124)

3. "inner ear"[Title/Abstract] OR "cochlea*"[Title/Abstract] OR "the organ of corti"[Title/Abstract] OR "endolymphatic duct"[Title/Abstract] OR "endolymphatic sac"[Title/Abstract] OR "organ of corti"[Title/Abstract] OR "vestibular organ"[Title/Abstract] OR "vestibular system"[Title/Abstract] (56852)

4. "macrophage*"[Text Word] OR "monocyte*"[Text Word] OR "leukocyte*"[Text Word] OR "lymphocyte*"[Text Word] OR "mast cell*"[Text Word] (997708)

5. #1 OR #3 (82359)

6. #2 OR #4 (1346277)

7. "immune cell*"[Text Word] OR "immunity"[Text Word] OR "resident cell*"[Text Word] OR "resident immune cell*"[Text Word] OR "inflammation cell*"[Text Word] OR "inflammatory cell*"[Text Word] (370936)

8. #5 AND #7 (275)

9. #5 AND #6 (660)

10. #8 OR #9 (845)

11. Limit #10 to English and German (782)

Database: Ovid MEDLINE (Literature search performed: August 22, 2022)

#	Searches	Results
1	Cochlea/	17924
2	Cochlea/cy, im [Cytology, Immunology]	1458
3	Ear, Inner/	13755
4	1 or 2 or 3	29670
5	Antibody-Producing Cells/	7418
6	Antigen-Presenting Cells/	12386
7	Leukocytes/	66878
8	Leukocytes, Mononuclear/	36743
9	bone marrow cells/ or granulocytes/ or hematopoietic stem cells/ or megakaryocytes/ or monocytes/	161349
10	Macrophages/	127892
11	Mast Cells/	28652
12	Myeloid Cells/	6011
13	Phagocytes/	8012
14	5 or 6 or 7 or 8 or 9 or 10 or 11 or 12 or 13	412981
15	inner ear.ti,ab,kw.	15672
16	cochlea*.ti,ab,kw.	42934
17	the organ of corti.ti,ab,kw.	3411
18	endolymphatic duct.ti,ab,kw.	357
19	endolymphatic sac.ti,ab,kw.	1548
20	organ of corti.ti,ab,kw.	3441
21	vestibular organ.ti,ab,kw.	354
22	vestibular system.ti,ab,kw.	3104
23	15 or 16 or 17 or 18 or 19 or 20 or 21 or 22	56919
24	monocyte*.tw.	110132
25	macrophage*.tw.	257281
26	leukocyte*.tw.	143295
27	lymphocyte*.tw.	328823
28	mast cell*.tw.	40404
29	24 or 25 or 26 or 27 or 28	744912
30	4 or 23	67185
31	14 or 29	922733

32	immune cell*.tw.	55686
33	immunity.tw.	179311
34	resident cell*.tw.	2162
35	resident immune cell*.tw.	815
36	inflammation cell*.tw.	1388
37	inflammatory cell*.tw.	42726
38	32 or 33 or 34 or 35 or 36 or 37	270657
39	30 and 38	222
40	30 and 31	480
41	39 or 40	645
42	limit 41 to (english or german)	595

Database: EBSCOhost CINAHL (Literature search performed: August 22, 2022)

#	Searches	Results
1	Cochlea/	20549
2	[Cochlea/cy, im [Cytology, Immunology]]	0
3	Ear, Inner/	18289
4	1 or 2 or 3	35763
5	Antibody-Producing Cells/	659
6	Antigen-Presenting Cells/	31708
7	Leukocytes/	116186
8	Leukocytes, Mononuclear/	42112
9	bone marrow cells/ or granulocytes/ or hematopoietic stem cells/ or megakaryocytes/ or monocytes/	263219
10	Macrophages/	209533
11	Mast Cells/	44193
12	Myeloid Cells/	77154
13	Phagocytes/	16242
14	5 or 6 or 7 or 8 or 9 or 10 or 11 or 12 or 13	642508
15	inner ear.ti,ab,kw.	20355
16	cochlea*.ti,ab,kw.	52611
17	the organ of corti.ti,ab,kw.	4378
18	endolymphatic duct.ti,ab,kw.	487
19	endolymphatic sac.ti,ab,kw.	1896
20	organ of corti.ti,ab,kw.	4520
21	vestibular organ.ti,ab,kw.	576
22	vestibular system.ti,ab,kw.	4552
23	15 or 16 or 17 or 18 or 19 or 20 or 21 or 22	71184
24	monocyte*.tw.	151671
25	macrophage*.tw.	336931
26	leukocyte*.tw.	193232
27	lymphocyte*.tw.	439808
28	mast cell*.tw.	53673
29	24 or 25 or 26 or 27 or 28	993095
30	4 or 23	81863
31	14 or 29	1256799

32	immune cell*.tw.	87249
33	immunity.tw.	234112
34	resident cell*.tw.	2925
35	resident immune cell*.tw.	1248
36	inflammation cell*.tw.	1995
37	inflammatory cell*.tw.	61031
38	32 or 33 or 34 or 35 or 36 or 37	370940
39	30 and 38	275
40	30 and 31	630
41	39 or 40	845
42	limit 41 to (english or german)	784

Database: LIVIVO (Literature search performed: August 22, 2022)

(((((((TI=(inner ear) OR KW=(inner ear)) OR TI=cochlea* OR KW=cochlea*) OR TI=(the organ of corti) OR KW=(the organ of corti)) OR TI=(endolymphatic duct) OR KW=(endolymphatic duct)) OR TI=(endolymphatic sac) OR KW=(endolymphatic sac)) OR TI=(organ of corti) OR KW=(organ of corti)) OR TI=(vestibular organ) OR KW=(vestibular organ)) OR TI=(vestibular system) OR KW=(vestibular system)

(((((((((((MESH=(Antibody-Producing Cells)) OR MESH=(Antigen-Presenting Cells)) OR MESH=Leukocytes) OR MESH=(bone marrow cells)) OR MESH=granulocytes) OR MESH=(hematopoietic stem cells)) OR MESH=megakaryocytes) OR MESH=monocytes) OR MESH=Macrophages) OR MESH=(Mast Cells)) OR MESH=(Myeloid Cells)) OR MESH=Phagocytes)

((((KW=monocyte*) OR KW=macrophage*) OR KW=leukocyte*) OR KW=lymphocyte*) OR KW=(mast cell*)

(((((KW=(immune cell*)) OR KW=immunity) OR KW=(resident cell*)) OR KW=(resident immune cell*)) OR KW=(inflammation cell*)) OR KW=(inflammatory cell*)

((((MESH=cochlea OR MESH=(inner ear)) OR (((((((TI=(inner ear) OR KW=(inner ear)) OR TI=cochlea* OR KW=cochlea*) OR TI=(the organ of corti) OR KW=(the organ of corti)) OR TI=(endolymphatic duct) OR KW=(endolymphatic duct)) OR TI=(endolymphatic sac) OR KW=(endolymphatic sac)) OR TI=(organ of corti) OR KW=(organ of corti)) OR TI=(vestibular organ) OR KW=(vestibular organ)) OR TI=(vestibular system) OR KW=(vestibular system))) AND (((((((((((((MESH=(Antibody-Producing Cells)) OR MESH=(Antigen-Presenting Cells)) OR MESH=Leukocytes) OR MESH=(bone marrow cells)) OR MESH=granulocytes) OR MESH=(hematopoietic stem cells)) OR MESH=megakaryocytes) OR MESH=monocytes) OR MESH=Macrophages) OR MESH=(Mast Cells)) OR MESH=(Myeloid Cells)) OR MESH=Phagocytes)) OR ((((KW=monocyte*) OR KW=macrophage*) OR KW=leukocyte*) OR KW=lymphocyte*) OR KW=(mast cell*))) OR (((MESH=cochlea OR MESH=(inner ear)) OR (((((((TI=(inner ear) OR KW=(inner ear)) OR TI=cochlea* OR KW=cochlea*) OR TI=(the organ of corti) OR KW=(the organ of corti)) OR TI=(endolymphatic duct) OR KW=(endolymphatic duct)) OR TI=(endolymphatic sac) OR KW=(endolymphatic sac)) OR TI=(organ of corti) OR KW=(organ of corti)) OR TI=(vestibular organ) OR KW=(vestibular organ)) OR TI=(vestibular system) OR KW=(vestibular system))) AND ((((((KW=(immune cell*)) OR KW=immunity) OR KW=(resident cell*)) OR KW=(resident immune cell*)) OR KW=(inflammation cell*)) OR KW=(inflammatory cell*))
Limit: German and English (386)
